# Inner ear exosomes and their potential use as biomarkers

**DOI:** 10.1371/journal.pone.0198029

**Published:** 2018-06-22

**Authors:** Eugene H. C. Wong, You Yi Dong, Mali Coray, Maurizio Cortada, Soledad Levano, Alexander Schmidt, Yves Brand, Daniel Bodmer, Laurent Muller

**Affiliations:** 1 Department of Otolaryngology, Head and Neck Surgery, University Hospital of Basel, Basel, Switzerland; 2 Department of Biomedicine, University of Basel, Basel, Switzerland; 3 Proteomics Core Facility, Biozentrum, University of Basel, Basel, Switzerland; Gustave Roussy, FRANCE

## Abstract

Exosomes are nanovesicles involved in intercellular communications. They are released by a variety of cell types; however, their presence in the inner ear has not been described in the literature. The aims of this study were to determine if exosomes are present in the inner ear and, if present, characterize the changes in their protein content in response to ototoxic stress. In this laboratory investigation, inner ear explants of 5-day-old Wistar rats were cultured and treated with either cisplatin or gentamicin. Hair cell damage was assessed by confocal microscopy. Exosomes were isolated using ExoQuick, serial centrifugation, and mini-column methods. Confirmation and characterization of exosomes was carried out using transmission electron microscopy (TEM), ZetaView, BCA protein analysis, and proteomics. Vesicles with a typical size distribution for exosomes were observed using TEM and ZetaView. Proteomic analysis detected typical exosome markers and markers for the organ of Corti. There was a statistically significant reduction in the exosome protein level and number of particles per cubic centimeter when the samples were exposed to ototoxic stress. Proteomic analysis also detected clear differences in protein expression when ototoxic medications were introduced. Significant changes in the proteomes of the exosomes were previously described in the context of hearing loss and ototoxic treatment. This is the first report describing exosomes derived from the inner ear. These findings may present an opportunity to conduct further studies with the hope of using exosomes as a biomarker to monitor inner ear function in the future.

## Introduction

Exosomes are membrane-bound nanovesicles, [1] with diameters ranging from 30–150 nm [2], that are released from both normal and diseased cells into interstitial and bodily fluids. [1]

The important roles of exosomes in physiological and pathological processes were frequently unrecognized due to their small size. [1] However, they are accepted as components of intercellular communication systems that can modulate their target cells’ function. [1] Research on the involvement of exosomes in disease has expanded over the past years, with studies focusing on their usage as biomarkers in the diagnosis, prognosis, and management of various diseases. [3]

However, to our knowledge, the presence of exosomes in the inner ear has not been described in the literature. One author described the presence of extracellular vesicles that resemble exosomes being released from a human vestibular Schwannoma cell line, [4] while another author found heat shock protein (HSP), a protein known to be present in exosomes, being released by the glial supporting cells in the inner ear. [5]

Exosome levels in the plasma or other bodily fluids increase during disease processes and decrease during recovery or after treatment. [6, 7] Whether the inner ear also produces exosomes under stressful conditions would be interesting and important to determine, as well as whether they play a role in sensorineural hearing loss (SNHL) induced by insults such as ototoxic medications and inflammation. Since exosomes provide molecular information about the parental cells that produce them, they can also potentially be used as biomarkers to help with diagnosis, disease monitoring, and prognosis of inner ear diseases.

The aims of the study were to show the presence of exosomes in the inner ear and characterize the changes in their protein content in response to ototoxic medications.

## Materials and methods

### Animal care

The animals used in this study were five-day-old Wistar rat pups (Harlan, Indianapolis, IN, USA) that were housed under pathogen-free conditions at the animal facility of the Department of Biomedicine of the University Hospital of Basel.

All procedures were conducted with the approval of the Animal Care Committee of Canton Basel City, Switzerland (Kantonales Veterinäramt Basel, Permit Number: 2263) in accordance with the European Communities Council Directive of 24 November 1986 (86/609/EEC).

### Preparation of inner ear cultures

All rat pups were decapitated and the whole inner ear (cochlea, vestibular organ, and neurons) was dissected from the skull in cold 1× phosphate buffered saline (PBS). The organ explants were then placed in culture medium (Dulbecco’s modified Eagle’s medium [DMEM]; Gibco by Invitrogen, Carlsbad, CA, USA) supplemented with 10% fetal bovine serum (FBS) (Sigma-Aldrich, Steinheim, Germany), 25 mM HEPES (Gibco), and 30 U/ml penicillin (Invitrogen). Explants were then incubated at 37 °C under 5% CO_2_, followed by recovery for 24 hours under the same conditions. The FBS used in this study was depleted of microvesicles by ultracentrifugation. Overall, we cultured 104 inner ear explants for this study.

The inner ears were then used as either controls (without treatment) or treated with cisplatin (Abcam, Cambridge, UK) or gentamicin (Sigma-Aldrich) at final concentrations of 160 μM and 50 μM, respectively, in the cell culture medium for 48 hours at 37°C under 5% CO_2_. Control organs were incubated for 48 hours in culture medium in DMSO only. Supernatants from the culture dishes were aspirated and placed in a new tube for exosome isolation.

### Isolation and confirmation of exosomes

#### Exosome isolation

The exosomes were initially isolated using ExoQuick-TC (EQ, System Biosciences Inc.; Mountain View, CA) according to the manufacturer’s recommended protocol. The medium was mixed with ExoQuick and incubated overnight (i.e., at least 12 hours) at 4°C. The samples were then centrifuged at 1500×g for 30 minutes, with removal of supernatant after centrifugation. A second centrifugation was performed at 1500×g for 5 minutes. The residual pellets were resuspended in 200 μL of PBS for analysis.

Once results confirmed the possible presence of exosomes in the samples, serial centrifugation and mini-column methods were used as described previously, [2] which is a validated technique known to produce highly purified exosomes. Using this method, the culture medium was centrifuged at 1000×g and 3000×g for 10 minutes each, followed by 10 000×g for 30 minutes, each done at 4°C. After each spin, sediments at the bottom of each tube were removed. The resultant supernatant was then passed through a 1.5 cm × 12 cm mini-column (Bio-Rad, Hercules, CA, USA; Econo-Pac columns) packed with Sepharose 2B (Sigma-Aldrich, St. Louis, MO, USA). The column bed volume was 10 ml. The column was washed with 20 ml of PBS and a porous frit was placed at the top of the gel to prevent any disturbance during subsequent elution with PBS. Fractions 1–3 (first 3 ml) were discarded, while #4 (1 ml) was collected and ultracentrifuged at 105.000xg for 2 hours. The supernatant was then discarded and the resultant pellets were suspended in 20–100 μl of PBS, depending on the size of the pellets.

#### Confirmation of exosomes

Transmission electron microscopy (TEM) (FEI/Philips CM200 FEG, Amsterdam, Netherlands) was performed on isolated exosomes after fixation on a 400-mesh square copper grid with 2% uranyl acetate using a negative staining method.

ZetaView (Particle Metrix GmbH, Meerbusch, Germany) was used to detect nano particles of the correct size and distribution or concentration of exosomes as recommended by the company.

Exosome protein levels were determined using the BCA Protein Assay Reagent kit (Pierce, Rockford, USA) according to the manufacturer’s instructions.

The exosome samples were also analyzed using a label-free quantitative mass spectrometry-based proteomics approach at the Proteomics Core Facility of the University of Basel, Switzerland, as recently described [8]. In brief, samples were dissolved in lysis buffer (1% sodium deoxycholate, 0.1M ammoniumbicarbonate), reduced with 5mM TCEP for 15 min at 95°C and alkylated with 10mM iodoacetamide for 30min in the dark at room temperature. Samples were diluted, digested with trypsin (Promega) at 37°C overnight (protein to trypsin ratio: 50:1) and desalted on C18 reversed phase spin columns according to the manufacturer’s instructions (Microspin, Harvard Apparatus).

1μg of peptides of each sample were subjected to LC–MS analysis using a dual pressure LTQ-Orbitrap Elite mass spectrometer connected to an electrospray ion source (both Thermo Fisher Scientific). Peptide separation was carried out using an EASY nLC-1000 system (Thermo Fisher Scientific) equipped with a RP-HPLC column (75μm × 30cm) packed in-house with C18 resin (ReproSil-Pur C18–AQ, 1.9μm resin; Dr. Maisch GmbH, Ammerbuch-Entringen, Germany) using a linear gradient from 95% solvent A (0.15% formic acid, 2% acetonitrile) and 5% solvent B (98% acetonitrile, 0.15% formic acid) to 28% solvent B over 75min at a flow rate of 0.2μl/min. The data acquisition mode was set to obtain one high resolution MS scan in the FT part of the mass spectrometer at a resolution of 240,000 full width at half-maximum (at m/z 400) followed by MS/MS scans in the linear ion trap of the 20 most intense ions. The charged state screening modus was enabled to exclude unassigned and singly charged ions and the dynamic exclusion duration was set to 20s. The ion accumulation time was set to 300ms (MS) and 50ms (MS/MS). The collision energy was set to 35%, and one microscan was acquired for each spectrum. For all LC-MS measurements, singly charged ions and ions with unassigned charge state were excluded from triggering MS2 events.

To determine changes in protein expressions across samples, a MS1 based label-free quantification was carried out. Therefore, the generated raw files were imported into the Progenesis QI software (Nonlinear Dynamics, Version 2.0) and analyzed using the default parameter settings. MS/MS-data were exported directly from Progenesis QI in mgf format and searched against a decoy database of the forward and reverse sequences of the predicted proteome from *rattus norvegicus* (Uniprot, download date: 24/03/2017, total of 72,508 entries) using MASCOT. The search criteria were set as following: full tryptic specificity was required (cleavage after lysine or arginine residues); 3 missed cleavages were allowed; carbamidomethylation (C) was set as fixed modification; oxidation (M) as variable modification. The mass tolerance was set to 10 ppm for precursor ions and 0.6 Da for fragment ions. Results from the database search were imported into Progenesis and the protein false discovery rate (FDR) was set to 1% using the number of reverse hits in the dataset. The final protein lists of all quantified peptides for each protein were exported and further statically analyzed using an in-house developed R script (SafeQuant, https://github.com/eahrne/SafeQuant). Two independent experiments were performed; (I) comparison two control, two Cisplatin- and one Gentamicin-treated samples and (II) comparison of control and Cisplatin-treated samples in biological triplicates. The results details of the two proteomics experiments carried out including identification scores, number of peptides quantified, normalized (by sum of all peak intensities) peak intensities, log_2_ ratios, coefficients of variations and p-values for each quantified protein and sample are displayed in S1 File. All raw data and results associated with the manuscript have been deposited in to the ProteomeXchange Consortium via the PRIDE [9] partner repository with the dataset identifier PXD009483 and 10.6019/PXD009483 (Reviewer account details: Username: reviewer11926@ebi.ac.uk, Password: of9Cfsth).

### Organ of Corti (OC) dissection and tissue culture

All animal experiments were carried out with the approval of the Animal Care Committee of the Canton of Basel, Switzerland. OCs from 5-day-old Wistar rat pups (Janvier Labs, Le Genest-Saint-Isle, France) were dissected from the skull and then placed in Dulbecco’s modified Eagle medium supplemented with 10% fetal bovine serum, 25 mM of HEPES, and 30 U/mL of penicillin (all from Sigma Aldrich Chemie GmbH, Steinheim, Germany). Explants were incubated at 37°C in 5% CO2. After 24h recovery, hair cell damage was induced by exposure to 50 μM gentamicin and 160 μM cisplatin for 48 h.

### Hair cell (HC) count

After treatment, the OCs were fixed, permeabilized, and stained with Alexa Fluor 568 phalloidin (Invitrogen AG, Basel, Switzerland). The images were acquired using the Nikon A1R laser confocal microscope with a × 20 lens (Nikon AG Instruments, Egg, Switzerland). The surviving HCs were counted in a section corresponding to 20 IHCs at different sites of the apical, basal and middle turn of each OC in three randomly selected fields. The inner hair cells (IHCs) and outer hair cells (OHCs) were counted to determine HC survival. If there was a gap in the normal ordered array of HCs, cells were considered to be missing because they had undergone apoptosis. The results are presented as the number of surviving hair cells per cochlear turn.

### Statistical analysis

The Statistical Package for the Social Sciences version 23 (IBM SPSS Statistics 23) and GraphPad Prism version 7.0 for Macintosh (La Jolla, California, USA) were utilized for statistical analysis. The comparisons between the exosome protein levels (mg/ml) in the control and treatment (cisplatin or gentamicin) groups were analyzed with Wilcoxon matched-pairs signed-rank test. On the other hand, the comparisons of particle concentration (×10^7^/cm^3^) between the control and treatment group were analyzed using the paired one-tailed student t test. A value of p<0.05 was considered significant. The paired tests were used since for each rat pup, we generated 2 matched pairs, 1 explant used as a control and 1 treated with ototoxic drugs. For the ototoxicity experiments a 2-way ANOVA and a Bonferroni’s multiple comparisons were used to analyze HC damage.

## Results

### Detection of inner ear exosomes

**Fig 1:**
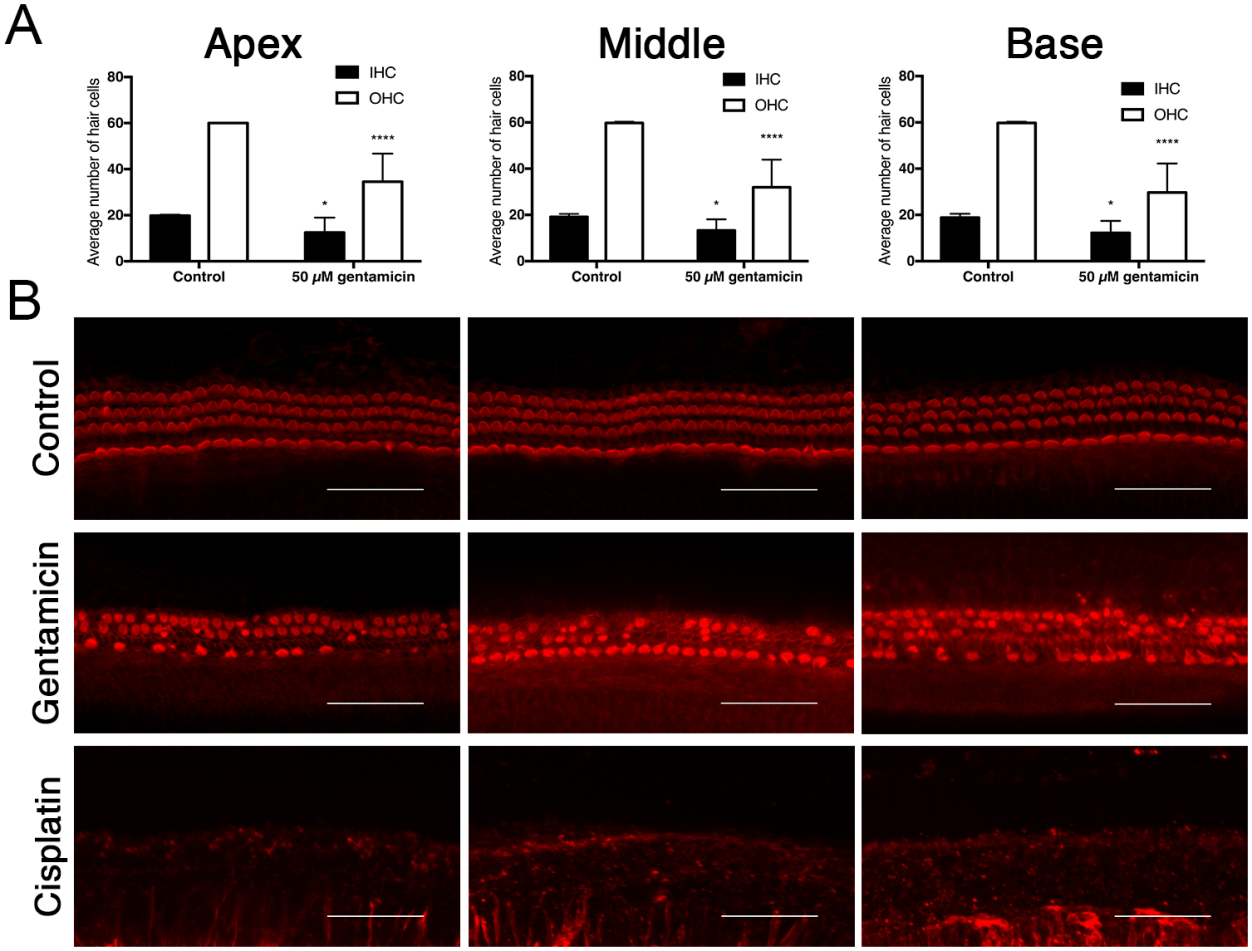
Gentamicin and cisplatin-induced ototoxicity. (A) Quantification of inner hair cells (IHCs) and outer hair cells (OHCs) in organ of Corti (OC) explants exposed to 50 μM gentamicin and 160 μM cisplatin for 48 hours. n = 6 OCs per condition. (B) Representative images of phalloidin-stained HCs. Gentamicin induced HC damage, cisplatin induced complete HC loss. Scale bar for all figures, 50 μm. Data are expressed as the number of surviving HCs per 20 IHCs, counted at different sites for the apical, middle and basal turn of each OC. Values are shown as means + SD. ****p < 0.0001 and *p < 0.05 versus untreated control group.

As a proof of concept for our experimental setup, we confirmed that the viability of the cells in the control group was good for up to 4 days. After ototoxic stress, the behavior of the isolated inner ear cells of the rat pups in our study were tested and found to be comparable to the previous experiments done in our laboratory; profound hair cell damage was caused by cisplatin and less so by gentamicin (Fig 1). [10–12] We then collected the supernatant from the control and intervention groups. The concentrations of cisplatin and gentamicin were selected according to established ototoxic concentrations from previous experiments. [11, 13] Exosomes were isolated from the supernatant at different time points and analyzed by different methods as indicated in S1 Fig.

**Fig 2:**
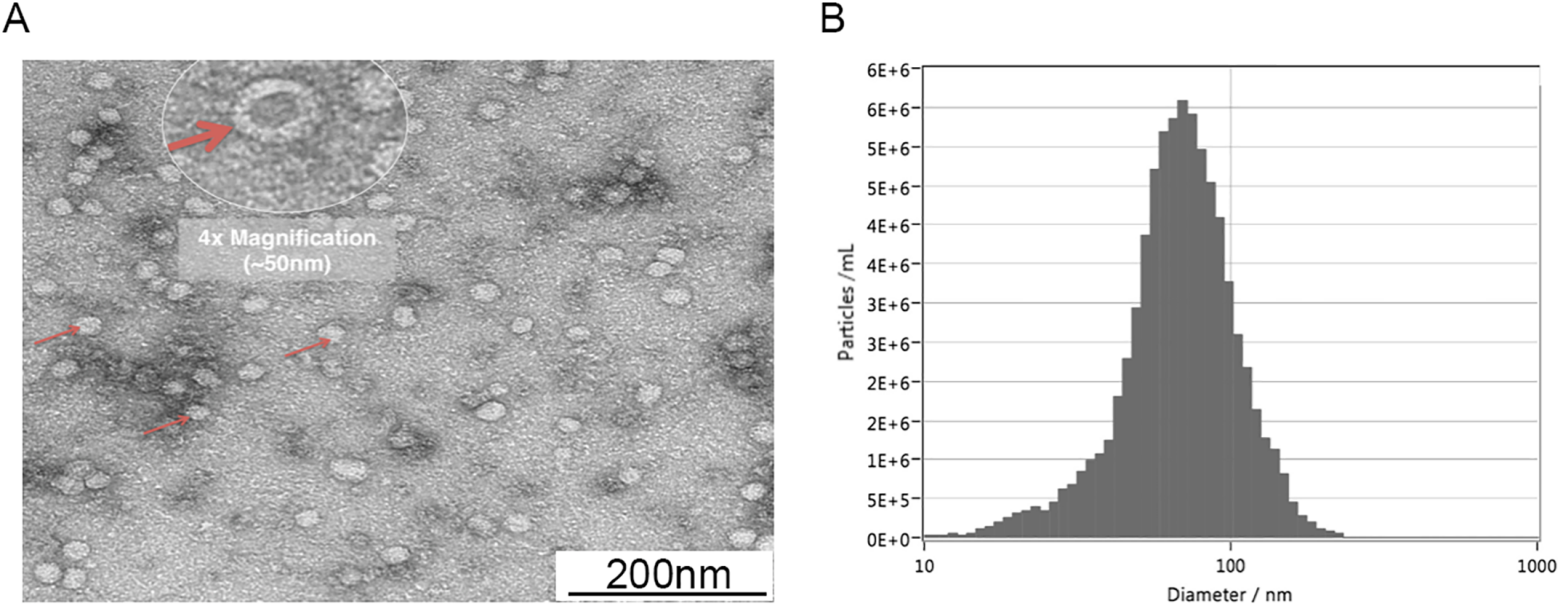
(A) Representative picture of several TEM studies showing the exosome vesicles, which had a typical size of 30–150 nm. When zoomed in (4× digital magnification), the uniform, cup-shaped morphology typical of exosomes was also observed. (B) Graph obtained from one of the exosome samples showing the average size distribution of the particles, which is typical of exosomes.

When isolated exosome preparations were visualized under TEM, we observed multiple extracellular vesicles, with sizes ranging from 30–150 nm that are typical of exosomes. When magnified, these vesicles have a uniform, cup-shaped appearance, which is the typical morphology of exosomes (Fig 2a).

Particles of the size distribution typical of exosomes were also observed when isolated vesicle samples were viewed with ZetaView (Fig 2b). The mean diameter of the vesicles in our sample was 109 ± 7.14 nm.

Furthermore, we also performed BCA assays to measure the mean exosome protein level. The average concentration of all the control samples was 23 μg/ml, which was clearly less than the concentration of exosomes typically found in cancer patients (mean of 50 μg/ml) [14].

Detection of typical exosome markers using Western blot, flow cytometry, and ELISA was not possible due to the low concentration of the protein yield, even after pooling samples in repeated attempts to increase the end sample concentration. Therefore, we instead performed proteomic analyses using highly sensitive mass spectrometry, where 454 proteins of rat origin were detected using this method. Typical exosome markers found included HSP 70/71 (Hspa5, Hspa1a, Hspa8) and HSP 90 (Hsp90b1, Hsp90aa1, Hsp90b1, Hsp90ab1). We also detected markers for the organ of Corti and hair cells such as alpha-actinin 1 (Actn 1) and Actn 4, myosin heavy chain (Mh9), and ATP5A1. Additionally, we also detected markers that are usually associated with SNHL such as myosin heavy chain (Myh 14), and markers associated with congenital SNHL and abnormal ear cartilage like V-type proton ATPase subunit B (V-ATPase B). We did not detect markers for other parts of the inner ear such as the vestibular organ, stria vascularis, or otoconial matrix.

### Inner ear exosomes as biomarkers of ototoxic stress

**Fig 3:**
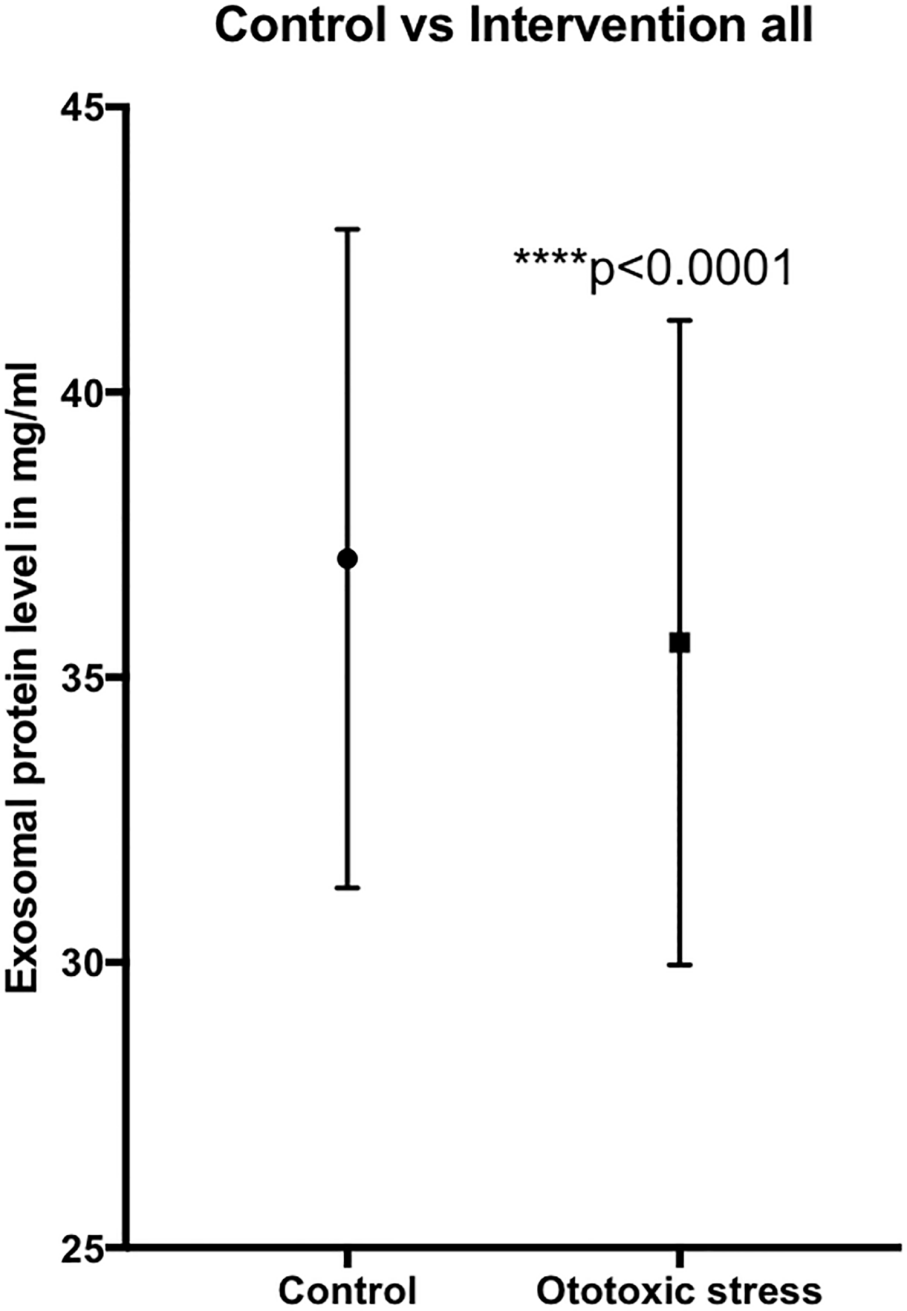
Comparison of mean (including standard error) exosome protein levels between the control group and samples treated with cisplatin or gentamicin using Wilcoxon matched-pairs signed-rank test, showing a statistically significant reduction in protein levels in the treated samples. ****p-value = 0.0001.

Overall, when comparing between control and all the samples (n = 76) treated with ototoxic medications (regardless of type), there was a statistically significant reduction in exosome protein levels (p<0.0001) (Fig 3). When analyzed separately, there was still a statistically significant reduction in the exosome protein levels between the control and samples treated with both cisplatin (n = 44) (p = 0.0014) and gentamicin (n = 20) (p = 0.0059) (S2 Fig).

**Fig 4:**
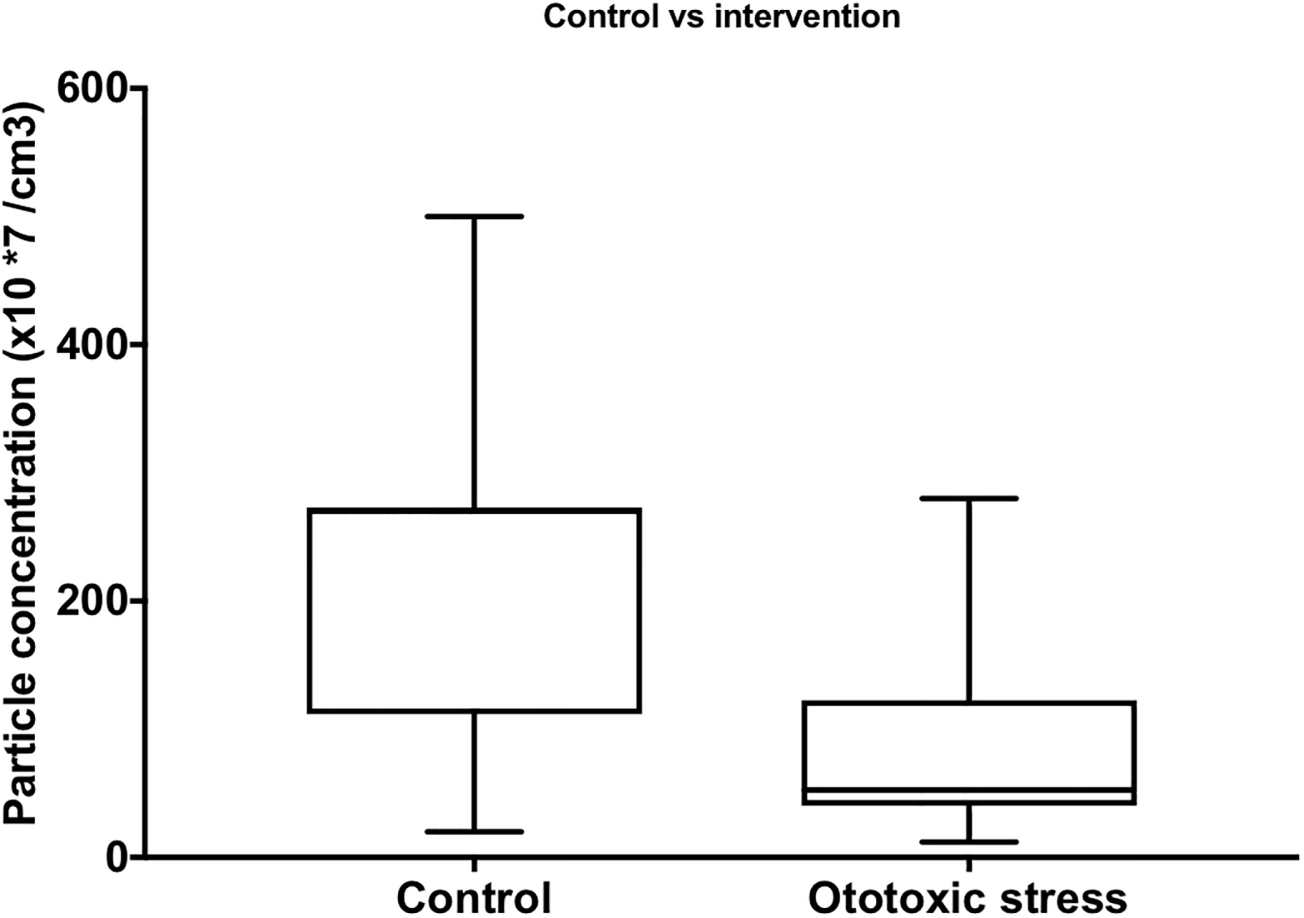
Comparison of the number of particles per cubic centimeter between the control group and samples treated with ototoxic medications using paired one-tailed student t test, showing a statistically significant reduction in the number of particles in the treated samples (p = 0.0012).

There was also a statistically significant reduction in the number of particles per cubic centimeter when ototoxic stressors were added to the samples (p = 0.0012) (Fig 4).

**Fig 5:**
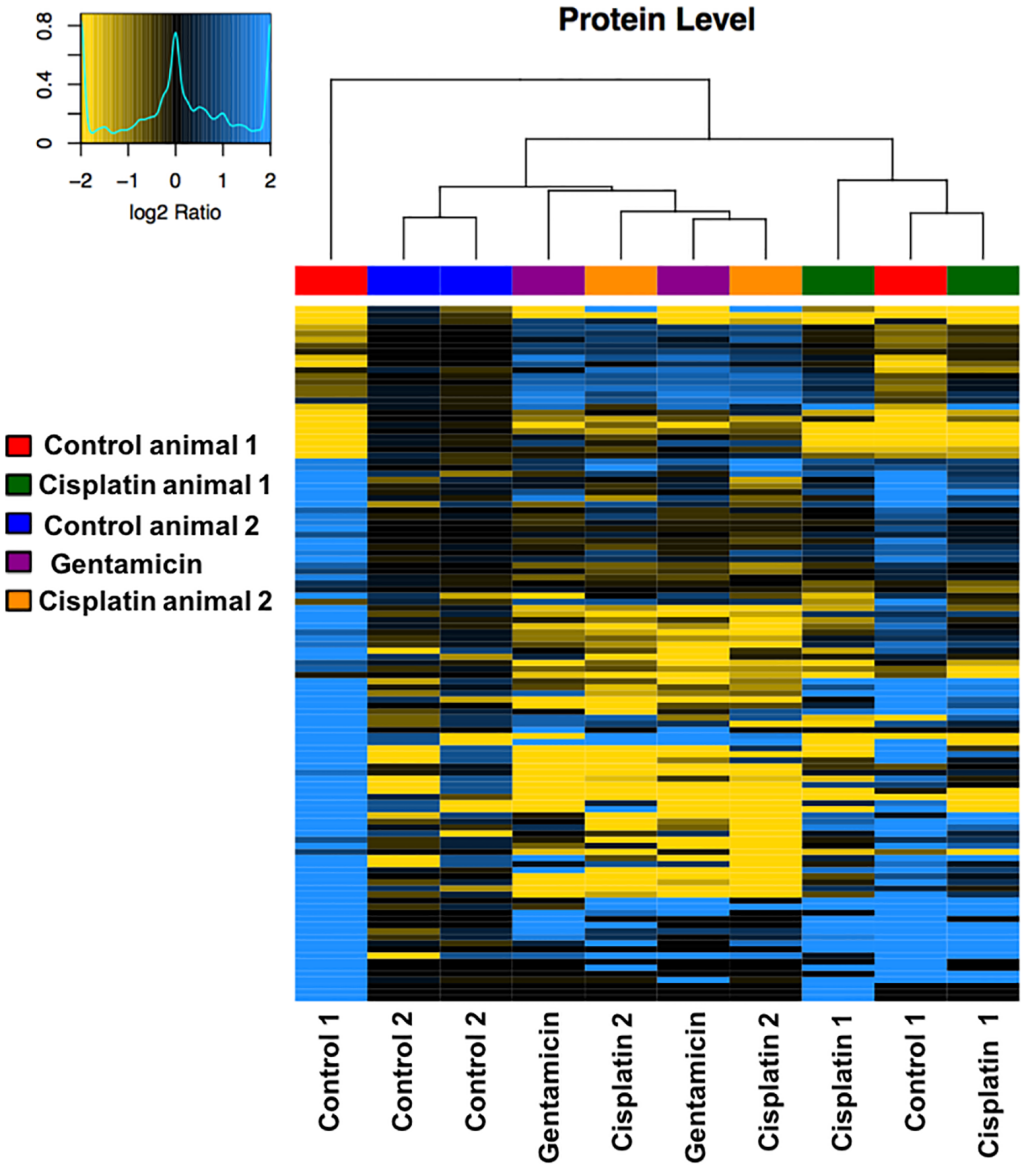
Representative heat map obtained after hierarchical clustering of protein log_2_ abundance ratios using Ward’s algorithm and the Pearson Correlation distance metric showing difference in protein expressions between various sample groups. The dendrogram illustrates similarity of protein abundance patterns. Control and Cisplatin treated samples were generated from two different animals (animal 1 and 2 as indicated) and two independent samples (from each ear) were analyzed per animal, respectively. For Gentamicin treatment, only two independent samples from one animal were analyzed. The black color represents centered median data with the blue representing up-regulation and yellow representing down-regulation of protein expression. Protein names and quantitative results are shown in S1 File.

From the proteomics analysis, there were also differences in protein expression patterns observed in samples treated with either cisplatin or gentamicin compared to the control group. Fig 5 is a heat map showing an overview of the protein expression patterns in different groups of samples. There were clear differences in protein expression between the control group and the cisplatin and gentamicin groups.

**Fig 6:**
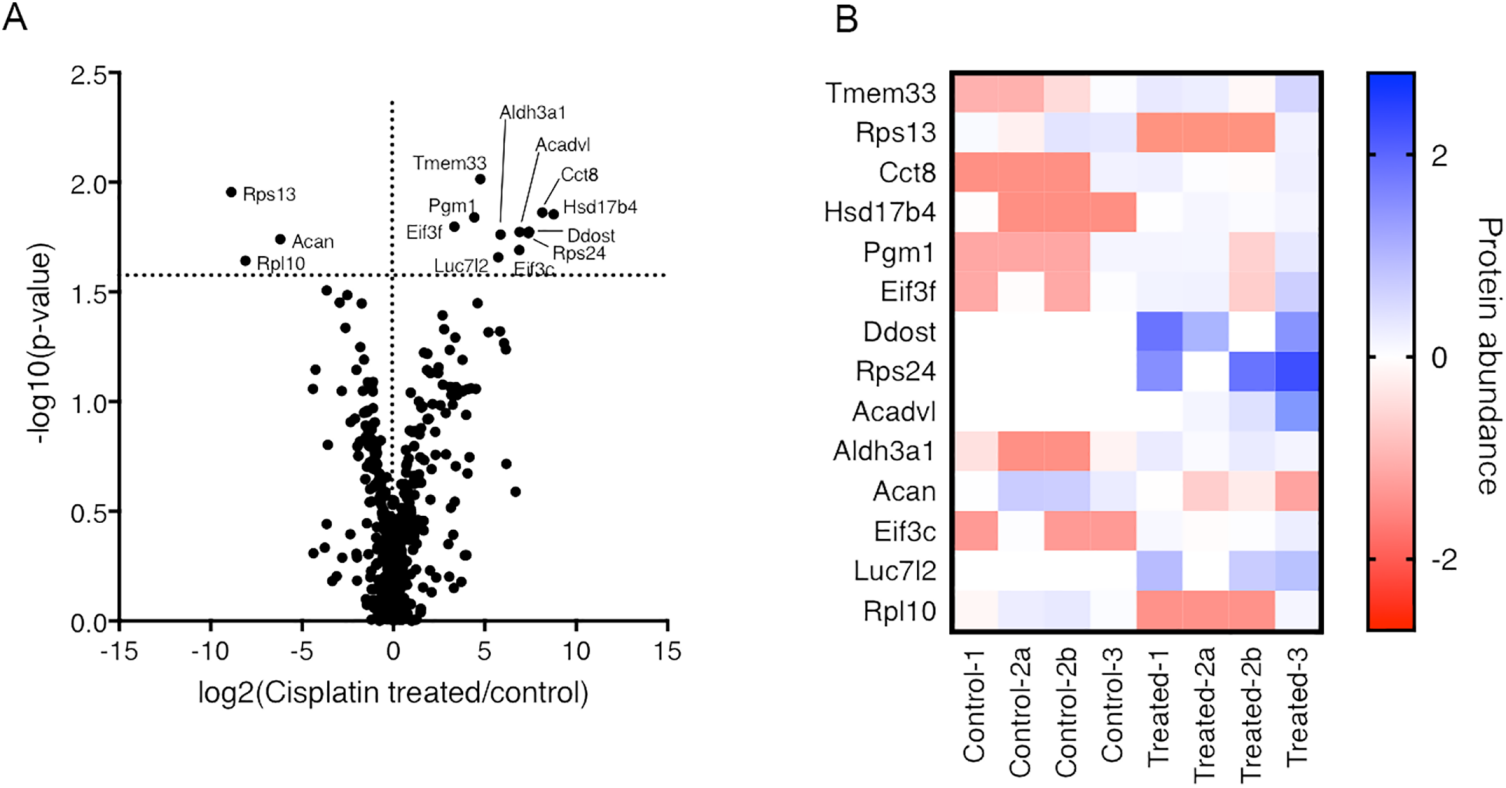
(A) Volcano Plot showing the −log_10_ p-values as a function of log_2_ ratios between control and Cisplatin treated samples analyzed in biological triplicates. Minus sign represents a decrease and plus sign an increase in protein expression from isolated inner ear exosomes. Two dashed black lines indicating no abundance change (log_2_ ratio = 0) and the p-value cut off (p<0.025) are indicated. (B) Heat map illustrating the abundances of the most significantly de-regulated proteins (as log_10_(MS-abundance(sample/median all samples)) found across the single samples analyzed. Sample 2 was analyzed in technical duplicate (a&b). The complete quantitative proteomics results are shown in S1 File.

Fig 6 illustrates the top hits that were significantly different between the control and intervention groups. For example, there were significant reductions in abundances of ribosomal protein S13 (Rps13), L10 (Rpl10) and Acan. Conversely, significant increases in expressions of Tmem 33, Pgm1, Eif3f, Rps24, Cct8, Hsd17b4, Aldh3a1, Ddost, Aldh3a1, Eif3c, Luc7l2 and Acadvl were observed for the intervention group.

## Discussion

Extracellular vesicles were isolated from cultured primary rat inner ear cells using an established technique that gives highly purified and functional exosomes. [2, 14] These isolated vesicles had typical characteristics (size range and appearance) for exosomes as seen with electron microscopy. When examined using ZetaView, these vesicles were around 100 nm.

Additionally, the potential use of exosomes as biomarkers was also tested in this ex vivo system. The ototoxicity effect of gentamicin and cisplatin on inner ear cells was tested using the same method as previously published by our group. [11, 13] As shown in Fig 1 profound hair cell damage was caused mostly by cisplatin and less so by gentamicin. We compared the exosome protein levels and particle concentrations in samples with and without gentamicin or cisplatin and noticed a reduction in both parameters in the group treated with ototoxic medications. The isolated exosomes were then subjected to proteomic analysis where proteins typically expressed in exosomes, as well as inner ear markers, were detected. Interestingly, the protein profile was strikingly different in the treated samples than in the control group, and proteins associated with sensorineural hearing loss were also seen. As such our most significant hits: Tmem33 [15] has been described in noise-traumatized rat cochleae, Pgm1 [16] in cisplatin-induced cytotoxicity and Cct8 [17] in aminoglykoside-induced cytotoxicity. Taken together exosomes levels not only decrease and their protein signature changes, but the exosomes in the treated group appears to enrich in certain proteins that may reflect the cell’s state.

Tmem33 (Transmembrane Protein 3) has been localized in the endoplasmatic reticulum (ER) and nuclear envelope. Its exact function needs to elucidated. It is thought that Tmem33 is involved in the regulation of the tubular structure of the ER because of its ability to interfere with the reticulons [18]. Possibly it could be an interesting target for future research.

To our knowledge this is the first report of the presence of exosomes in the inner ear. The presence of exosomes in the inner ear was to be expected since they are known to be released by various cell types in different body fluids and under several stressful conditions such as hypoxia, [19] heat shock, [20] oxidative stress, [21] acidic pH, [22] and cancer. [23] They are also involved in normal physiological processes such as exporting waste products and eliminating drugs. [1] The exact role of exosomes is still unknown, although they are known to play important roles in intercellular communication, [24] genetic material exchange, angiogenesis, immune modulation, tumor metastasis, and oncogene distribution. [25]

A number of limitations were identified in our study. For example, we used inner ears from 5-day-old rats’ for all our studies. Exosome release may differ in rats of different ages or in humans. We also used a fixed concentration of cisplatin and gentamicin based on our established laboratory data that we have shown has a negative impact on inner ear cells. Different drug concentrations may also have different effects. Furthermore, we cultured the organ of Corti as a whole and did not separate it into the various parts. Another obstacle faced in this study was the low concentration of exosomes yielded from each culture, where we typically only get 20 μg of proteins from 1 ml of culture medium. This low concentration of protein made it impossible to further confirm the presence of exosomes using classic methods such as Western blot, mass spectrometry, or ELISA, all of which were attempted many times, even by using pooled samples to increase the end sample concentration.

These findings are very important, especially as a starting point for further research. If their presence is proven in human ears, the exact role of exosomes in the inner ear needs to be deciphered. The detection of exosomes and changes in their protein profile may be of interest in the treatment of otologic pathologies, especially of the inner ear, where they could also be used to monitor inner ear damage secondary to ototoxic medications to help guide clinical therapy accordingly, or in patients with sudden SHNL. Further research in this field is required, although the potential use of exosomes as biomarkers seems evident.

## Conclusion

We found exosomes present in the inner ear, which are most likely produced by the organ of Corti. We also found a statistically significant reduction in exosome protein levels and the number of particles per cubic centimeter when ototoxic stress was introduced to the exosomes. This may be translated by the reduced cell number, since heavy hair cell damage occurs especially in the cisplatin group. Differences in protein expression patterns were also detected in the group treated with ototoxic drugs when compared to the control group. The interesting finding is that the significant hits in the proteomics analyses of the exosomes have previously been described in the context of hearing loss (especially Tmem33) and as such exosomes are not only changing in number and protein compositions, but seem to reflect the inner ear hair cells status. This qualifies exosomes as ideal candidates to be used as biomarkers, also more work needs to be done to further characterize the inner ear exosomes as well as to determine their exact role in the inner ear, especially when ototoxic medications are administered.

## Supporting information

S1 Fig This illustrates the flow chart of the study methodologies. (TIFF)

S2 Fig Comparisons of exosomal protein level between control and samples treated with gentamicin (left graph) and cisplatin (right graph) using Wilcoxon matched-pairs signed-rank test, showing statistically significant reduction of protein level in the treated sample. ** p-value = 0.0059 (left) and **p-value = 0.0014 (right).
(TIFF)

S1 File Table representing the complete proteomics results. (XLSX)
