# Supplementary figures and images for: Inner ear exosomes and their potential use as biomarkers
Source: PLoS One. 2018 Jun 22;13(6):e0198029. doi: 10.1371/journal.pone.0198029 (PMC6014643; doi:10.1371/journal.pone.0198029)

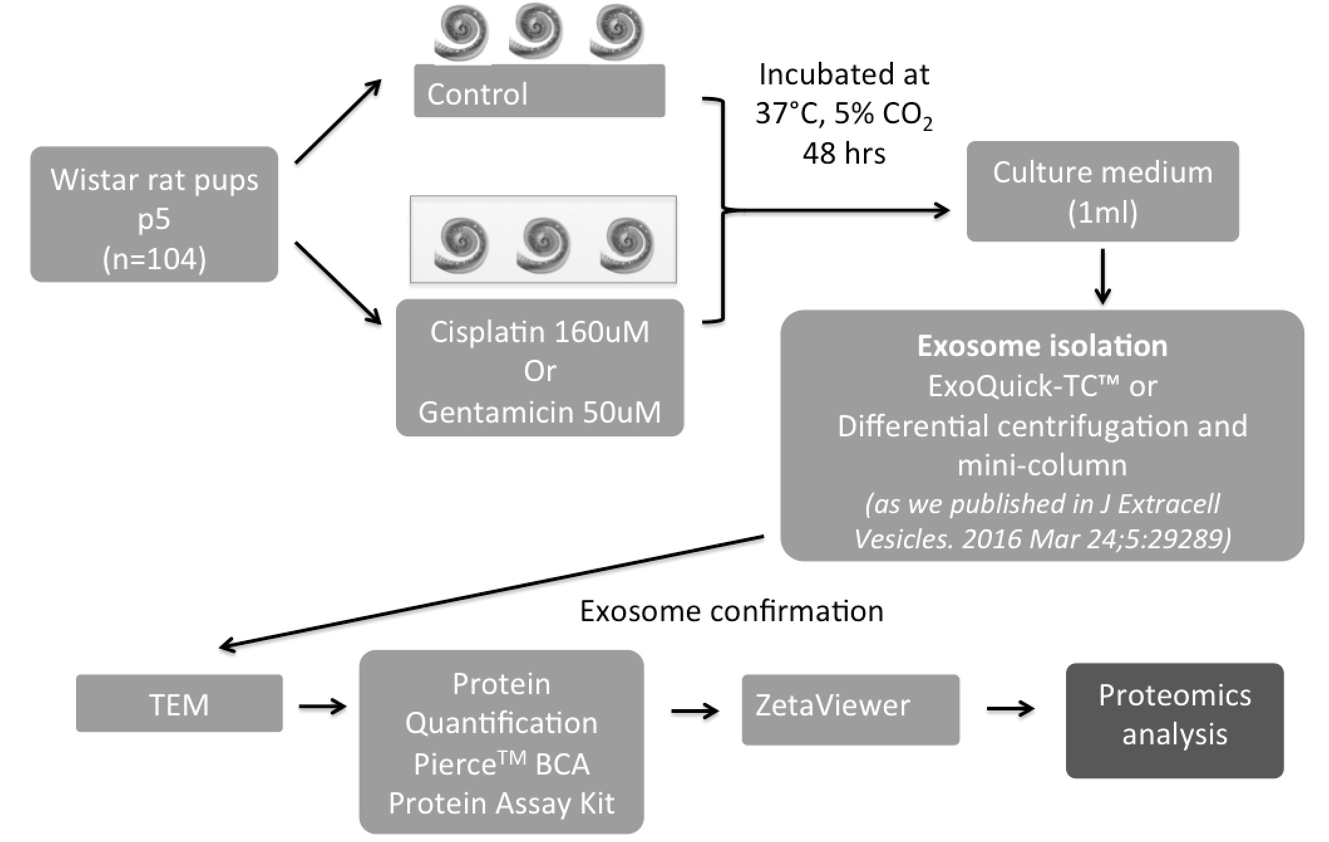

Supplement: S1 Fig — (TIFF) [file pone.0198029.s001.tiff]

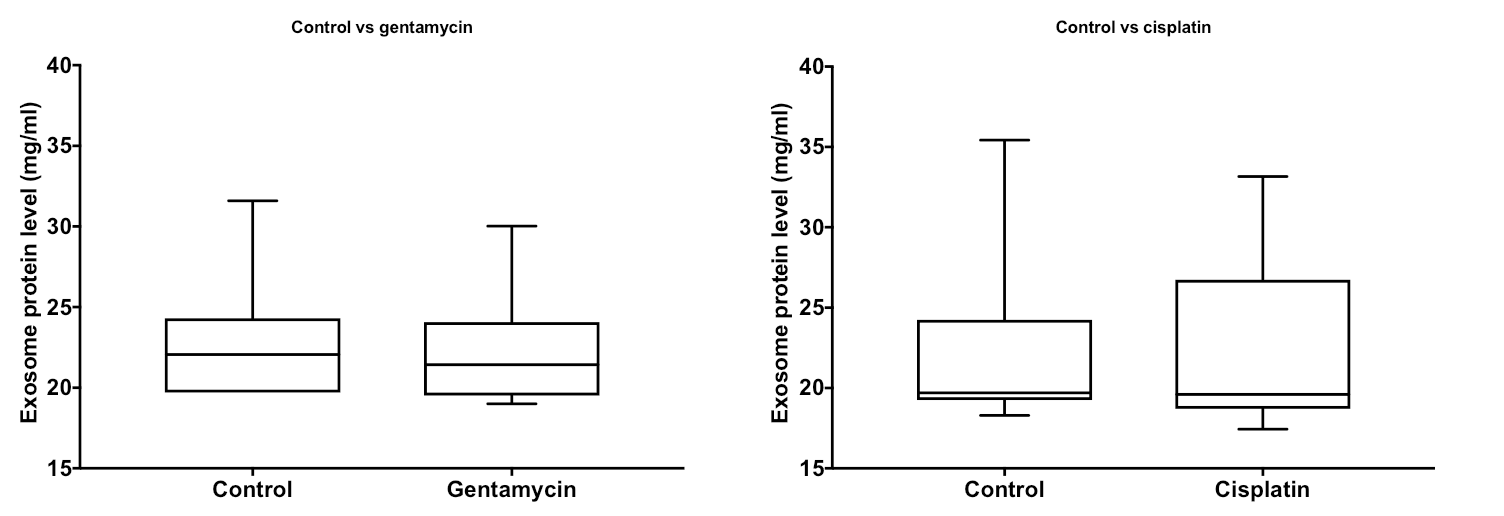

Supplement: S2 Fig — ** p-value = 0.0059 (left) and **p-value = 0.0014 (right). (TIFF) [file pone.0198029.s002.tiff]
